# Cyclin B1 Suppresses Colorectal Cancer Invasion and Metastasis by Regulating E-Cadherin

**DOI:** 10.1371/journal.pone.0126875

**Published:** 2015-05-11

**Authors:** Yifeng Fang, Xiao Liang, Wenbin Jiang, Jianbo Li, Junfen Xu, Xiujun Cai

**Affiliations:** 1 The Second Department of General Surgery, Sir Run Run Shaw Hospital, School of Medicine, Zhejiang University, Hangzhou, 310016, China; 2 Department of Gynecologic Oncology, Women’s Hospital, School of Medicine, Zhejiang University, Hangzhou, 310006, China; The University of Hong Kong, CHINA

## Abstract

Cyclin B1, a mitotic cyclin, has been implicated in malignances. However, its contribution to colorectal cancer invasion and metastasis are still not well understood. Here, we demonstrated that the invasion and metastasis of colorectal cancer is regulated by Cyclin B1. Overexpression of Cyclin B1 was observed in colorectal cancer tissues, but this elevated expression was negatively associated with lymph node metastasis, distant metastasis stage, and TNM stage. The Kaplan-Meier survival analysis proved that low Cyclin B1 expression was associated with poor overall survival of patients with colorectal cancer. Inhibition of Cyclin B1 in colorectal cancer cells enhanced the cell migration and invasion of three different colorectal cancer cell lines. In studying the possible mechanism by which Cyclin B1 suppresses colorectal cancer invasion and metastasis, we observed that suppression of Cyclin B1 decreased the expression of E-cadherin protein level. Our findings suggest that Cyclin B1 could suppress the invasion and metastasis of colorectal cancer cells through regulating E-cadherin expression, which enables the development of potential intervention strategies for colorectal cancer.

## Introduction

Despite increased general awareness, colorectal cancer remains one of the three leading causes of malignancy-related mortality worldwide [1]. The majority of the colorectal cancer patients in the early stage (TNM stage I and II) can be treated effectively by surgical resection and the 5-year survival rates for the early stage cancers is up to 95% (stage I) and 60–80% (stage II), respectively. However, most patients with metastatic colorectal cancer in the advanced stage (TNM stage III and IV) are usually refractory to existing therapies and have quite a poor prognosis since the 5-year survival rates drop dramatically to 35% with lymph node involvement (stage III) and to 10% when the disease has spread to distant organs (stage IV) [2–5]. Thus, metastasis formation is the decisive and the most lethal event during this disease course. In fact, tumor metastasis is a complicated biological process that involves primary tumor angiogenesis, cancer cell invasion, vascular/lymphatic intravasation, distant target organ extravasation, and the growth of invaded cells in foreign microenvironment to form metastatic colonization [6–8]. Although dysregulation of signaling pathways and dysfunction of many molecules have been identified in cancer metastasis, they cannot fully explain the phenomenon of colorectal cancer metastasis [2]. Thus, it is of great importance to uncover the biological mechanisms underlying metastasis in colorectal cancer and formulate strategies to intervene in this process.

Epithelial-mesenchymal transition (EMT) is known as a crucial mechanism in colorectal cancer metastasis and a key regulator of distant organ formation [9–11]. During the process of EMT, epithelium-derived tumor cells loss of cell adhesion and polarity, and gain of migratory and invasive properties, resulting in enabling tumor cells to infiltrate surrounding tissues, and thus licensing these cells to metastasize into distant tissues [9, 12]. At the molecular level, E-cadherin is the best-characterized molecular marker of EMT. Loss of E-cadherin expression is known as the most predominant hallmark of EMT [13–15]. E-cadherin, encoded by the CDH1 gene which is located on chromosome 16q22 [16], is a transmembrane glycoprotein confined to epithelial cells and is mainly responsible for intercellular adherence junctions [17]. Many transcription factors, such as Snail, ZEB1, ZEB2, FOXC2, Slug, and Twist have been shown to directly or indirectly cause the repression of E-cadherin promoter activity [18–21]. Besides, the loss or reduction of E-cadherin expression was found to permit or accelerate invasion and metastasis, and thus suggested as a potential prognostic factor in colorectal cancer [22–25].

Cyclin B1, mapped to human chromosome 5q12, is known as a mitotic cyclin because of its key role in modulating G2/M phase progression of the cell cycle, and participates in cell growth, differentiation, apoptosis, and metastasis in various cancer types [26–29]. So far, increased expression of Cyclin B1 has been reported in breast, prostate, esophageal, lung, colon, and gastric cancers [30–36]. In our previous study, we also found overexpression of Cyclin B1 promoted cell proliferation and tumor growth in human colorectal cancer [37]. However, absence of prognostic relevance or favorable prognosis of Cyclin B1 was also observed in colorectal cancer, lymphoma and pancreatic neuroendocrine tumor [35, 38, 39]. These discrepancies indicate further study to be needed.

In this study, to elucidate whether and how Cyclin B1 is involved in the cell invasion and metastasis in colorectal cancer, we initially evaluated Cyclin B1 expression in 150 pairs of colorectal cancer and matched adjacent non-tumor colorectal tissues, then analyzed its correlation with clinicopathological features. Interestingly, we found that overexpression of Cyclin B1 in colorectal cancer was negatively correlated with lymph node metastasis, distant metastasis, TNM stages, and poor survival. Our study also revealed inhibition of Cyclin B1 suppressed the expression of E-cadherin, and subsequently led to the induction of migration and invasion of colorectal cancer cells. These findings not only associate Cyclin B1 with metastasis in colorectal cancer, but also provide a promising target for treating the late stage patients with colorectal cancer.

## Materials and Methods

### Patients and specimens

Tumorous and matched adjacent normal colorectal tissues were obtained from 150 patients who underwent surgery at Sir Run Run Shaw Hospital, School of Medicine, Zhejiang University (Hangzhou, China), between April 2005 and June 2009. In particular, adjacent normal tissues were taken > 5 cm laterally from the edge of the cancerous region. None of these patients had received chemotherapy or radiotherapy prior to operation. The present study was approved by the Sir Run Run Shaw Hospital Research Ethical Committee and all the samples were obtained with written informed consent (S1 Fig). All specimens were immediately frozen into liquid nitrogen after surgical resection and stored at -80°C until RNA extraction.

Clinical information of these patients including age, gender, differentiation status, tumor location, tumor size, lymph node metastasis, distant metastasis, and TNM stage, was collected and shown in S1 Table. The patients were followed-up once every 3 months for at least 5 years postoperatively. All patients were contacted by phone or questionnaire letters to check upon their health status. Death of the patients was ascertained by report from their family.

### RNA extraction and quantitative real-time PCR

Total RNA was extracted using TRIzol reagent (Invitrogen) according to the manufacturer’s instructions. cDNA was synthesized from 2 ug total RNA using PrimeScript RT reagent Kit (TaKaRa) and amplified with SYBR Premix Ex Taq (TaKaRa) on ABI 7500HT system (ABI). The primer of Cyclin B1 was showed previously [37]. GAPDH was used as internal control. Relative quantification of mRNA expression was calculated with the 2^-ΔCT^ or 2^-ΔΔCT^ method.

### Cell culture and transfection

The human colorectal cancer cell lines p53^+/+^ HCT116, p53^-/-^ HCT116, and SW480 were obtained from the American Type Culture Collection. p53^+/+^ HCT116 and p53^-/-^ HCT116 cells were cultured in McCoy’s 5A complete medium (Gibco). The SW480 cells were cultured in RPMI 1640 medium supplemented with 10% FBS (Gibco). All the three cell lines were cultured in a humidified chamber with 5% CO_2_ at 37°C.

siRNA against Cyclin B1 and siRNA negative control were synthesized by bioneer. Cells were transiently transfected with siRNA using lipid-based transfection reagents DharmaFECT1 (Dharmacon) according to the manufacturer’s protocol. The transfection efficiency and transfection level of Cyclin B1 siRNA was determined previously [37].

### Cell migration assay

24h post-transfection, 1×10^5^ treated cancer cells in 200 μl of serum-free medium were added to the upper portion of the Transwells (8-μm pore size; Costar). The bottom chambers were filled with medium supplemented with 10% FBS and incubated at 37°C / 5% CO_2_ until the detection time (24h for p53^+/+^ HCT116, 36h for p53^-/-^ HCT116, and 36h for SW480, respectively). Cells on the underside of the membrane were fixed, stained with 0.1% crystal violet, and counted (five independent microscopic fields at a 20-fold magnification). Assays was performed in duplicate and repeated three independent times.

### Cell invasion assay

A 24-well transwell plate (8-μm pore size coated with matrigel (BD Biosciences) was used to measure cell invasion capacity. 24h after transfection, 1×10^5^ cells were resuspended in serum-free medium and added to the upper chamber. The medium supplemented with 10% FBS was used as chemoattractant to the lower chamber before examination. Cells were incubated at 37°C for the indicated time (30h for p53^+/+^ HCT116, 42h for p53^-/-^ HCT116, and 48h for SW480, respectively). The cells on the upper surface were scraped with cotton swabs and washed away, whereas the invaded cells on the lower surface were fixed, stained with 0.1% crystal violet for 1h, and quantified by determining the cell number in five randmonly chosen visual fields Assays was performed in duplicate and repeated three independent times.

### Western blot analysis

Cells were collected and lysed in RIPA buffer containing protease inhibitor (Pierce). Protein concentration was determined with BCA assay kit, and equal amount of protein were electrophoresed on 10% sodium dodecyl sulfate-polyacrylamide gel and transferred onto polyvinylidene difluoride membrances (PVDF, Millipore). After blocking for nonspecific binding, the membrane was incubated with specific primary antibodies against Cyclin B1 (1:2000; Epitomics), E-cadherin (1:1000; Abcam), and GAPDH (1:2,000; Santa Cruz) overnight at 4°C, and then washed three times with Tris-buffered saline and Tween 20, followed incubated with the appropriate horseradish peroxidase-conjugated secondary antibodies (HRP, Dawen Biotec) for 1h at room temperature. The protein expression was finally visualized using enhanced chemiluminescence detection (ECL, Biological Industries). Intensities of the protein expression signals were quantified using densitometric analysis with Quantity One software (Bio-Rad), and the resulting values of the protein were normalized to the corresponding loading controls.

### Immunohistochemistry

Anti-E-cadherin antibody (Epitomics) was used for IHC staining of the xenograft tumors. IHC was carried out as previously described [37].

### Statistical analysis

Statistical analysis was performed using SPSS standard version 21.0 (SPSS Inc) and GraphPad Prism 5 (GraphPad Software). Difference between groups in tissues samples was assessed by the nonparametric Mann-Whitney *U* test. Relative quantification of Cyclin B1 expression was calculated with the 2^-ΔCT^ or 2^-ΔΔCT^ method (ΔCT = Cyclin B1 (CT)—GAPDH (CT); ΔΔCT = ΔCT (tumor)- ΔCT (Normal)), and data are presented as mean ± SEM. Kaplan-Meier and log-rank tests were used to evaluate patient overall survival (OS) and to create survival curves based on the high and low Cyclin B1 levels (as defined by the ROC curve). The univariate and multivariate survival analysis were performed with the Cox regression model. All in vitro experiments were performed in duplicate and repeated three times. For the in vitro and in vivo animal experiments, difference between two groups was evaluated using the Student’s *t*-test, and data are presented as mean ± SD. Value of *P* < 0.05 was considered statistically significant.

## Results

### Cyclin B1 overexpression is negatively correlated with lymph node matastasis, distant metastasis and advanced TNM stage in human colorectal cancer tissues

**Fig 1:**
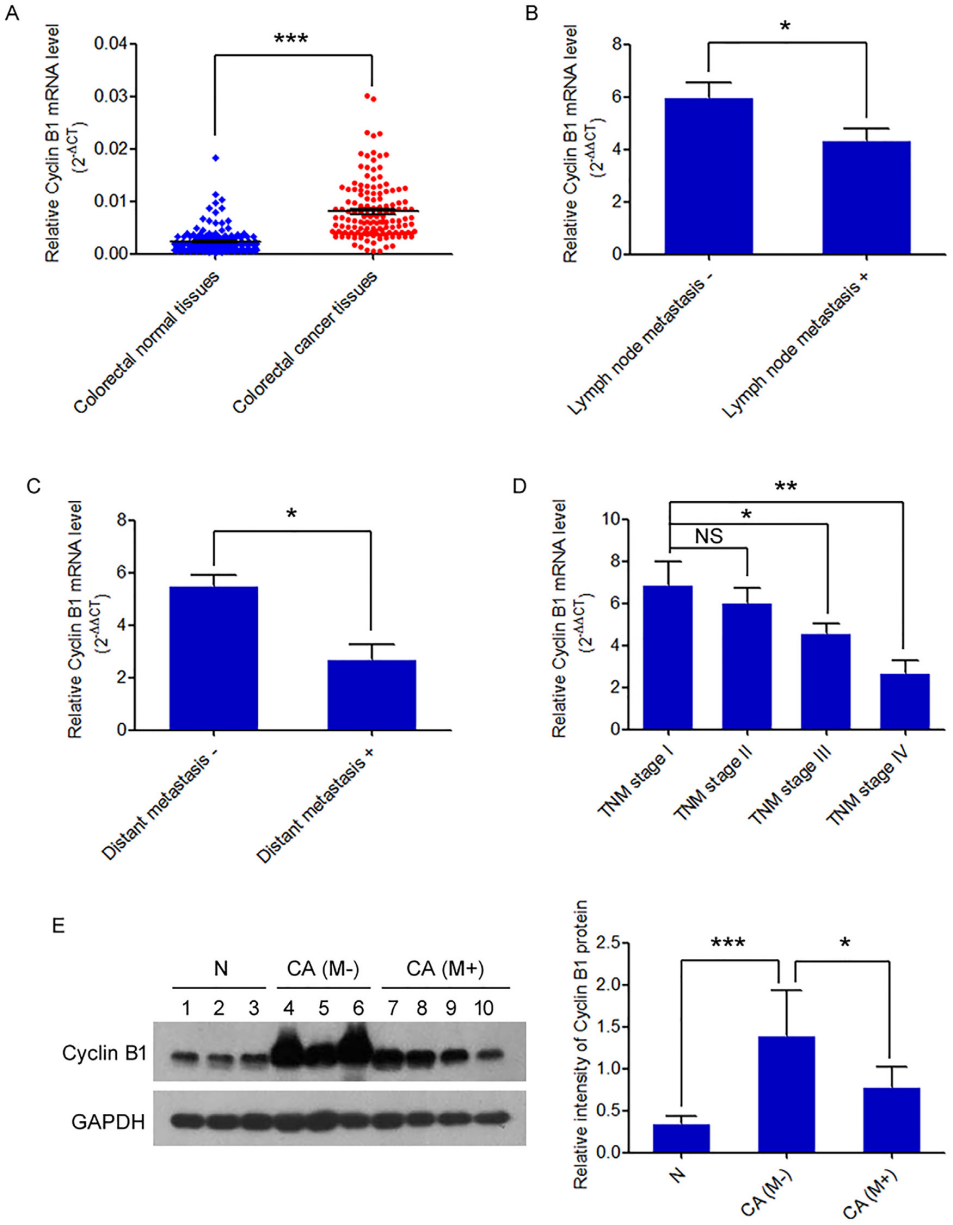
Cyclin B1 expression is increased in colorectal cancer tissues and negatively correlates with metastasis. (A) Cyclin B1 mRNA expression was tested using qRT-PCR in 150 pairs of human colorectal cancer tissues and adjacent non-tumorous tissues. Alterations in expression are shown as scatter diagrams, with the y-axis indicating Cyclin B1 expression. Its expression was normalized to the level of GAPDH expression in each sample. Data are presented as mean ± SEM. *** *P <* 0.001. (B) qRT-PCR analysis of Cyclin B1 expression in colorectal cancer patients with or without lymph node metastasis. The data are represented as a 2^-ΔΔCT^ (tumor/normal). * *P* < 0.05. (C) Statistical analysis of relative Cyclin B1 expression level in colorectal cancer tissues with or without distant metastasis. Data are presented as mean ± SEM. * *P* < 0.05. (D) Assessment of relative Cyclin B1 expression level in colorectal cancer tissues of TNM stage I, II, III, and IV. Data are presented as mean ± SEM. * *P* < 0.05; ** *P* < 0.01; NS, not significant. (E) Western blot validation of Cyclin B1 protein level in normal colorectal tissues (N) and colorectal cancer tissues (CA) with metastasis (M+) and without (M-). GAPDH was applied for normalization. Relative intensity of Cyclin B1 protein was shown in the following bar graphs. Data are presented as mean ± SD. *** *P* < 0.001. * *P* < 0.05.

To identify the potential role of Cyclin B1 in colorectal cancer metastasis, we initially examined the expression of Cyclin B1 in 150 pairs of colorectal cancers and matched adjacent non-tumor colorectal tissues using qRT-PCR. Cyclin B1 mRNA was overexpressed in 139/150 (92.7%) pairs, with an average increased fold change of 5.30 in cancer tissues. We further found that the relative mean expression of Cyclin B1 was much lower in patients with lymph node metastasis than that in patients without metastasis (*P* = 0.011) (Fig 1B). In patients with distant metastasis, the level of Cyclin B1 was also significantly lower than that in patients without distant metastasis (*P* = 0.021) (Fig 1C). As shown in Table 1, the association between Cyclin B1 level and clinicopathological characteristics showed the expression of Cyclin B1 was negatively correlated with lymph node metastasis, distant metastasis, and advanced TNM stage (*P* = 0.007) (Fig 1D) in patients with colorectal cancer. However, no significant association was found between Cyclin B1 expression and other clinicopathological features, such as patient age, gender, tumor site, tumor size and differentiation (all *P* > 0.05). Moreover, we also confirmed the protein level of Cyclin B1 in 30 clinical tissues samples, including 10 normal colorectal tissues, 10 colorectal cancer tissues without metastasis, and 10 cancer tissues with metastasis by western blot. We obtained consistent result with the RNA level result. Cyclin B1 protein was overexpressed in colorectal cancer tissues without metastasis, compare to the normal colorectal tissues (*P* = 5.51×10^–5^), whereas the Cyclin B1 protein level significantly decreased in colorectal cancer tissues with metastasis, when compared to those without metastasis (*P* = 0.012) (Fig 1E). These data reveal that the Cyclin B1 expression was inversely associated with colorectal cancer metastasis.

**Table 1:** Clinicopathological characteristics and Cyclin B1 expression in colorectal cancer patients.

Clinicopathological factors	Number of cases (n = 150) (%)	Median expression of CCNB1 (range) (2^-ΔΔCT^ ± SD)	*P* value
**Gender**			0.257^a^
Male	88 (58.7%)	5.12 ± 5.00 (0.027–39.295)	
Female	62 (41.3%)	5.56 ± 4.01 (0.069–17.175)	
**Age (years)**			0.443^a^
< 65	72 (48.0%)	4.81 ± 3.50 (0.027–17.175)	
≥ 65	78 (52.0%)	5.76 ± 5.41 (0.618–39.295)	
**Tumor site**			0.995^a^
Colon	77 (51.3%)	5.61 ± 5.47 (0.027-39-295)	
Rectum	73 (48.7%)	4.98 ± 3.47 (0.069–16.015)	
**Histology**			0.689^a^
Adenocarcinoma	136 (90.7%)	5.41 ± 4.76 (0.069–39.295)	
Mucinous	14 (9.3%)	4.29 ± 2.54 (0.027–8.681)	
**Tumor size (cm)**			0.187^a^
< 5	86 (57.3%)	5.43 ± 3.71 (0.069–16.015)	
≥ 5	64 (42.7%)	5.13 ± 5.61 (0.027–39.295)	
**Differentiation**			0.514^b^
Well	72 (48.0%)	5.45 ± 3.89 (0.069–17.175)	
Moderate	50 (33.3%)	5.08 ± 3.69 (0.898–14.529)	
Poor, mucinous	28 (18.7%)	5.32 ± 7.23 (0.027–39.295)	
**Local tumor invasion**			0.429^b^
T1	3 (2.0%)	6.27 ± 3.00 (3.847–9.627)	
T2	19 (12.7%)	6.07 ± 3.96 (0.458–13.631)	
T3	54 (36.0%)	5.35 ± 5.70 (0.027–39.295)	
T4	74 (49.3%)	5.03 ± 3.91 (0.069–17.175)	
**Lymph node metastasis**			**0.011** ^a^
Negative	88 (58.7%)	5.97 ± 5.20 (0.069–39.295)	
Positive	62 (41.3%)	4.35 ± 3.42 (0.027–13.576)	
**Distant metastasis**			**0.021** ^a^
Negative	139 (92.7%)	5.51 ± 4.70 (0.458–39.295)	
Positive	11 (7.3%)	2.70 ± 1.89 (0.027–5.987)	
**TNM stage**			**0.007** ^b^
I	15 (10.0%)	6.88 ± 4.19 (0.458–13.631)	
II	65 (43.3%)	6.04 ± 5.59 (0.747–39.295)	
III	59 (39.3%)	4.57 ± 3.47 (0.583–13.576)	
IV	11 (7.3%)	2.70 ± 1.89 (0.027–5.987)	

^a^ *P* value when expression levels were compared using the Mann—Whitney test

^b^ *P* value when expression levels were compared using the Kruskal- Wallis test

### Cyclin B1 expression is negatively associated with decreased survival in colorectal cancer

**Fig 2:**
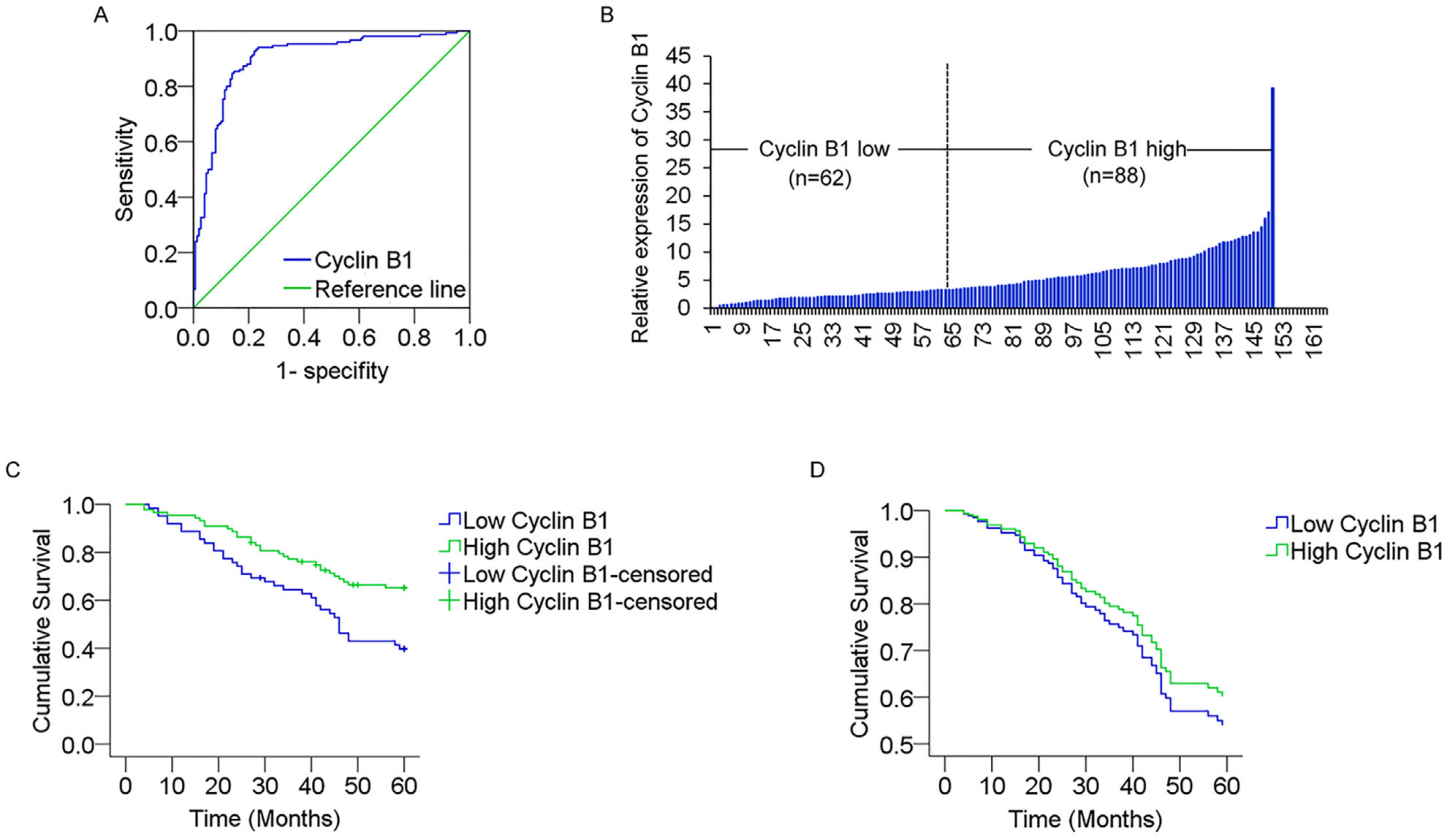
Cyclin B1 high expression negatively correlates with poor survival of colorectal cancer patients. (A) The optimal cut-off level of Cyclin B1 relative expression was determined based on the largest of sensitivity and specificity by receiver operating characteristic curve (ROC) analysis for overall survival. Area under curve (AUC) of ROC was 0.90. (B) According to the optimal cut-off value (3.33-fold), 150 patients with colorectal cancer were divided into two groups: ≥ 3.33 were considered as an elevated Cyclin B1 expression group (n = 88); < 3.33 were considered as a low Cyclin B1 expression group (n = 62). (C) Kaplan-Meier survival analysis according to Cyclin B1 expression in patients with colorectal cancer (log-rank test). (D) Cumulative overall survival curves of patients with high or low Cyclin B1 expression levels by multivariate analysis.

To evaluate the prognostic potential of Cyclin B1 in colorectal cancer, we analyzed the association between Cyclin B1 expression and survival duration using Kaplan-Meier analysis with log-rank test. We initially plotted the receiver operating characteristic (ROC) curve for Cyclin B1 (Fig 2A). According to the maximal Youden index, the optimal cut-off value for Cyclin B1 expression was 3.33 fold in tumor/non-tumor. Then the 150 patients with colorectal cancer were divided into two groups: high Cyclin B1 expression group (n = 88) that Cyclin B1 expression ratio ≥ 3.33, and low Cyclin B1 expression group (n = 62) that Cyclin B1 expression ratio < 3.33, according to the cut-off level (Fig 2B). Kaplan-Meier estimation analysis revealed a correlation between Cyclin B1 expression level and overall survival times. Patients with a low level of Cyclin B1 had an obviously lower survival rate than those with a high level of Cyclin B1 expression (*P* = 0.002) (Fig 2C).

To determine whether Cyclin B1 expression is an independent indicator of overall survical for colorectal cancer patients, we first used a univariate Cox proportional hazards regression model to estimate the individual hazard ratio for all of the clinicopathological characteristics. The results revealed that overall survival was significally related to the Cyclin B1 expression level (RR = 2.062; 95% CI, 1.273–3.340; P = 0.003) and other four parameters (tumor size, lymph node metastasis, distant metastasis, and TNM stage; all *P* < 0.05). However, in the multivariate analysis, the Cox proportional hazards model including Cyclin B1 expression, tumor size, lymph node metastasis, and distant metastasis, identified tumor size, lymph node metastasis and distant metastasis as the independent prognostic indicators for the overall survival rate of patients with colorectal cancer (HR:0.564, P = 0.022; HR:0.205, P = 2.0×10^–8^; HR: 0.148, P = 1.0×10^–6^, respectively), but not Cyclin B1 expression (p = 0.45) (Fig 2D). The statistical values of Cyclin B1 expression and other clinicopathological characteristics derived from the Cox proportional hazards regression model are showed in Table 2.

**Table 2:** Univariate and multivariate analysis of overall survival in colorectal cancer patients.

Clinicopathologic feature	Univariate analysis			Multivariate analysis		
	HR	95% CI	*P* value	RR	95% CI	*P* value
**Gender**	0.633	0.382–1.048	0.076			
**Age**	0.656	0.403–1.071	0.092			
**Tumor site**	1.310	0.809–2.121	0.272			
**Histology**	0.612	0.292–1.281	0.193			
**Tumor size**	0.499	0.308–0.808	**0.005**	0.564	0.345–0.922	**0.022**
**Differentiation**	0.643	0.333–1.066	0.187			
**Local tumor invasion**	0.791	0.473–1.325	0.373			
**Lymph node metastasis**	0.197	0.116–0.335	**1.80E-08**	0.205	0.118–0.357	**2.00E-08**
**Distant metastasis**	0.183	0.090–0.373	**3.00E-06**	0.148	0.069–0.318	**1.00E-06**
**TNM stage**	0.095	0.042–0.218	**2.70E-08**			
**cyclin B1 expression**	2.062	1.273–3.340	**0.003**	1.215	0.733–2.013	0.451

### Suppression of Cyclin B1 facilitated migratory ability in different colorectal cancer cells

**Fig 3:**
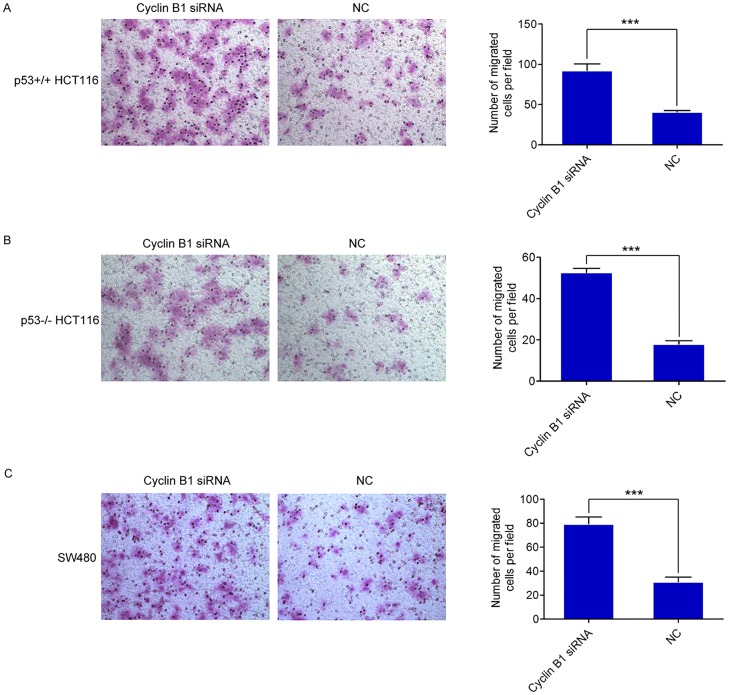
Inhibition of Cyclin B1 promotes the migration ability of colorectal cancer cells. (A, B, C) Transwell migration assays of p53^+/+^ HCT116, p53^-/-^ HCT116, and SW480 cells were performed after transfection with the Cyclin B1 siRNA or negative control, respectively. Representative images of migration assay were shown. In all panels, the results are representative of at least three independent experiments. NC, siRNA negative control. Data represent the average of three independent experiments ± SD. *** *P <* 0.001.

The above observations prompted us to explore the potential biological function of Cyclin B1 deregulation on colorectal cancer progression. The ability of a tumor cell to undergo migration allowed it to change the position within the tissues, and this process allows tumor cells to leave from their primary tumor [40]. Thus, we assessed the effect of Cyclin B1 on migration in colorectal cancer cells using Transwell assay. Three colorectal cancer cell lines, p53^+/+^ HCT116, p53^-/-^ HCT116, and SW480 were transfected with specific Cyclin B1 siRNA or siRNA negative control, respectively, as previously reported. The results demonstrated that inhibition of Cyclin B1 prominently enhanced the migratory capability of p53^+/+^ HCT116 cells by 127% (p = 1.45×10^–5^) (Fig 3A). Similarly, the migration capacity of p53^-/-^ HCT116 was also remarkably increased by 193% (p = 3.30×10^–8^) (Fig 3B) after the suppression of Cyclin B1 expression. Moreover, the migration activity in Cyclin B1 siRNA-transfected SW480 cells increased significantly by 155% (p = 2.85×10^–6^) (Fig 3C), compared to the control cells. These data indicate Cyclin B1 exerts a migration-suppressing function in human colorectal cancer cells and this effect is independent of cell type.

### Knockdown of Cyclin B1 promotes colorectal cancer cell invasion

**Fig 4:**
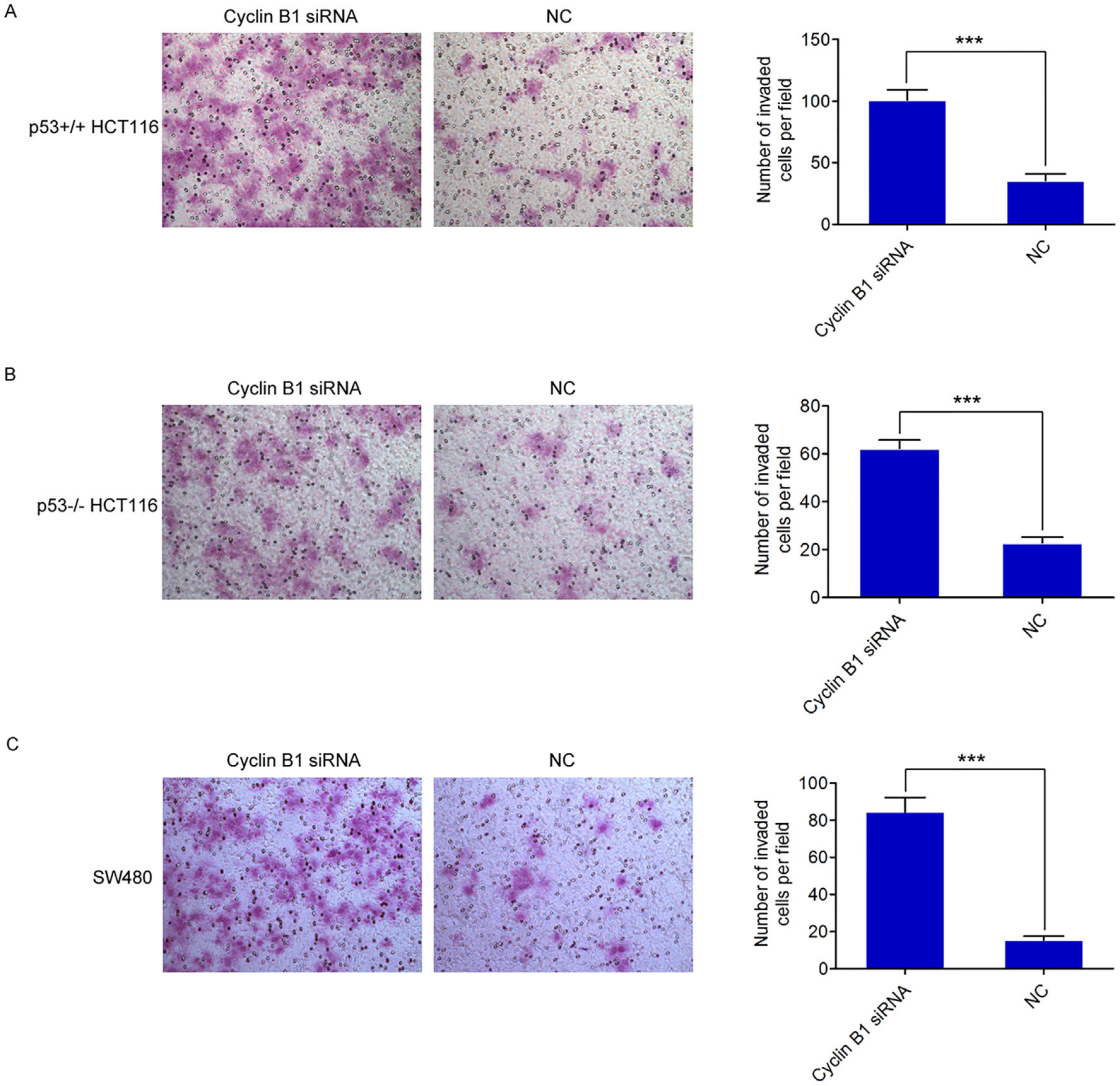
Suppression of Cyclin B1 facilitates the invasion ability of colorectal cancer cells. (A, B, C) The effect of Cyclin B1 on cell invasion was detected in three colorectal cancer cell lines, p53^+/+^ HCT116, p53^-/-^ HCT116, and SW480 cells, using transwell chamber assays. The chambers were coated with matrigel, which functions as extracellular cell matrix. Representative images of invaded cells are shown. The data are representative of three independent experiments. Error bars represent SD. *** *P <* 0.001.

Cell invasion is an intrinsic cellular process whereby cells respond to extracellular stimuli to move through and degrade the extracellular matrix (ECM). In cancer, cell invasion allows cancer cells to acquire the ability to enter lymphatic and/or blood vessels for dissemination into the circulation, followed by invasion to distant organs for metastatic growth [8, 40]. To investigate the potential effect of Cyclin B1 on cell invasion in colorectal cancer cells, we used the Matrigel Invasion assay for detection. We found that depletion of Cyclin B1 resulted in a 1.82-fold and 1.72-fold increase in the number of invading cells in both p53^+/+^ HCT116 and p53^-/-^ HCT116 cells (p = 6.88×10^–6^; p = 6.70×10^–6^, respectively) (Fig 4A and 4B). Likewise, inhibition of Cyclin B1 also increased the invasion activity of SW480 cells by 4.47-fold (p = 2.07×10^–7^) (Fig 4C). Collectively, these results suggest Cyclin B1 inhibits invasion of colorectal cancer cells.

### Inhibition of Cyclin B1 decreases E-Cadherin level in colorectal cancer cell lines

**Fig 5:**
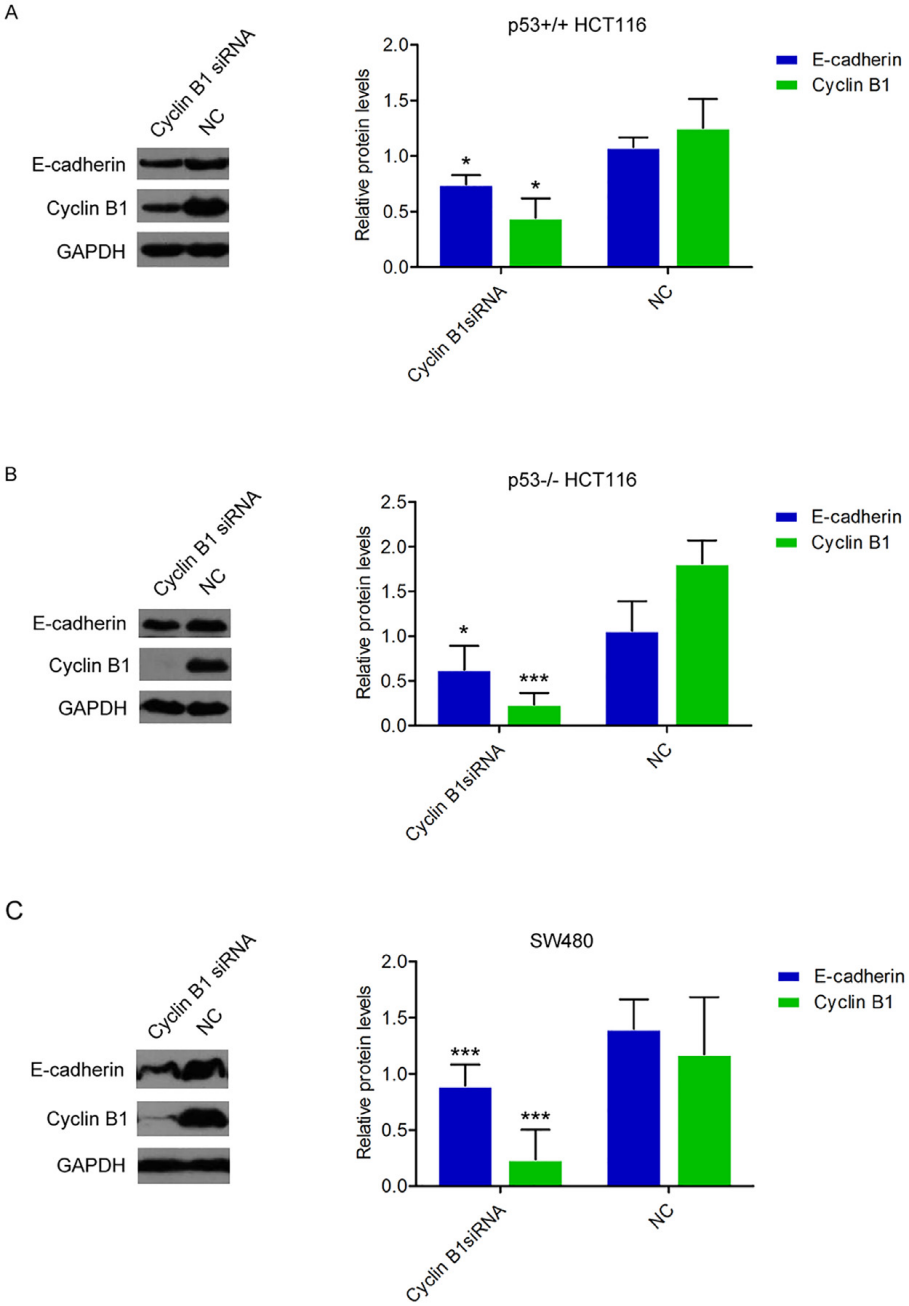
Cyclin B1 regulates E-cadherin expression level. (A, B, C) 72h after transfection with 50 nM of Cyclin B1 siRNA or siRNA negative control, the endogenous protein levels of E-cadherin and Cyclin B1 in p53^+/+^ HCT116, p53^-/-^ HCT116, and SW480 cells were analyzed by western blotting and quantified. Bars show the relative protein levels that are normalized to GAPDH. Data represent the average of three independent experiments ± SD. * *P <* 0.05; *** *P <* 0.001.

Given that Cyclin B1 negatively correlated with enhanced colorectal cancer cell migration and invasion, we examined whether EMT is an underlying mechanism. Dysregulation of E-cadherin has been considered to be the major critical molecule in EMT-mediated cancer cell metastasis. Here, we explored the role of Cyclin B1 in the regulation of E-cadherin expression in the above three colorectal cancer cell lines. Fig 5A and 5B both showed that Cyclin B1 siRNA transfection in p53^+/+^ HCT116 and p53^-/-^ HCT116 strongly decreased E-cadherin protein expression level, respectively (p = 0.038; p = 0.011, respectively). Also, in Cyclin B1 siRNA-treated SW480 cells, E-cadherin protein expression was obviously reduced compared with the expression in negative control-transfected cells (p = 0.0006) (Fig 5C). Our data indicate that Cyclin B1-mediated inhibition of cell invasion may occur via regulating E-cadherin expression.

### Inhibition of Cyclin B1 downregulates E-Cadherin expression in vivo

**Fig 6:**
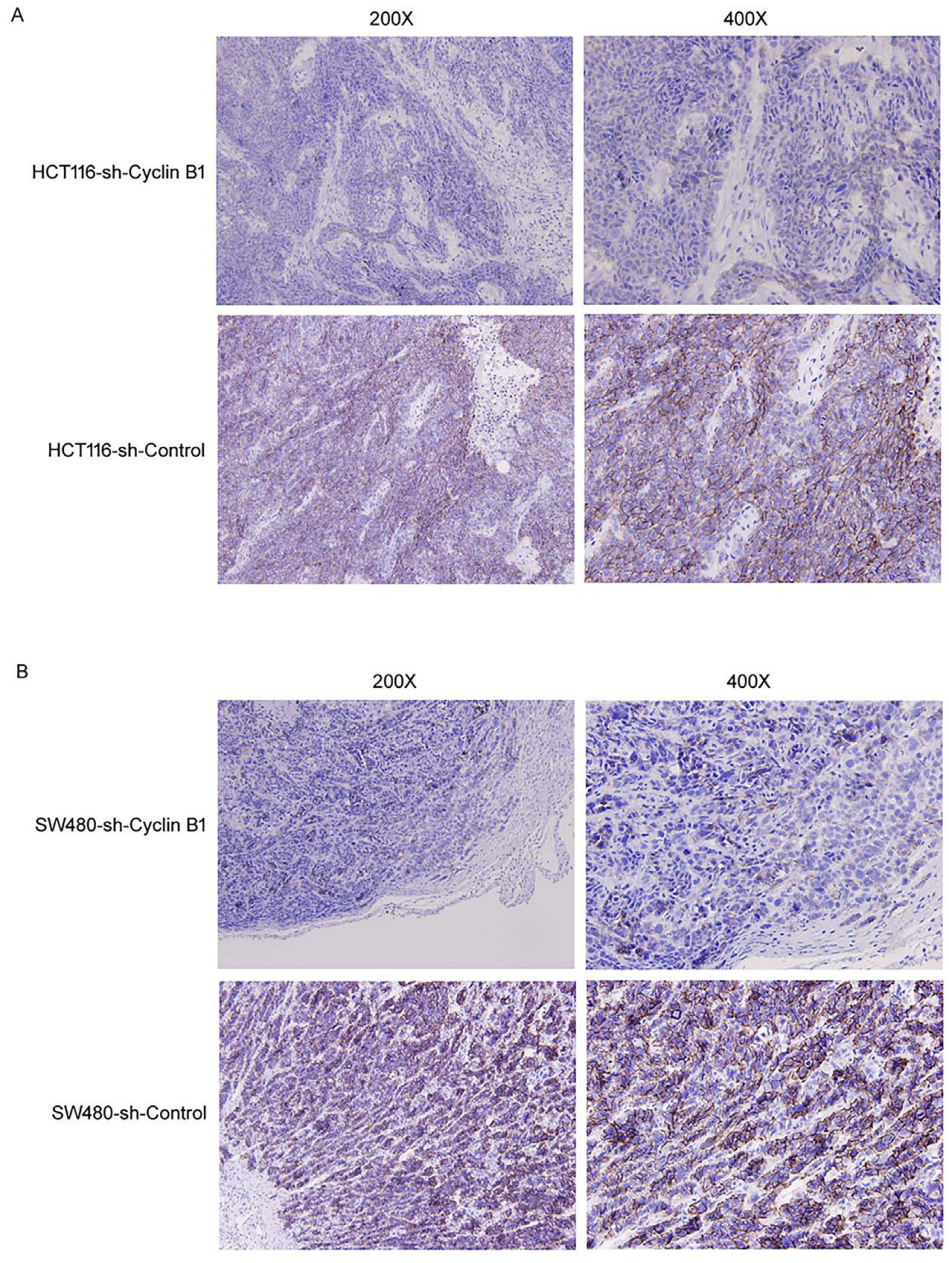
Representative immunohistochemical staining of E-cadherin in xenograft tumor. (A) Images (x200; x400) representing immunohistochemical staining of E-cadherin in p53^+/+^ HCT116 tumors expressing Cyclin B1 shRNA or vector control. (B) Represent images (x200; x400) of E-cadherin in SW480 tumors expressing Cyclin B1 shRNA or vector control.

To determine whether Cyclin B1 alters E-cadherin expression in vivo, we evaluated E-cadherin expression in our previous tumor xenograft models [37] by immunohistochemical staining. As shown in Fig 6A, E-cadherin level was significantly decreased in Cyclin B1-suppressing p53^+/+^ HCT116 tumors, compared to the Cyclin B1 non-suppressing control tumors. Similarly, E-cadherin protein was also obviously downregulated in Cyclin B1-suppressing SW480 tumors, compared to the control tumors (Fig 6B). These data further suggest the effect of Cyclin B1 on E-cadherin expression.

## Discussion

The major causes of colorectal cancer-related mortality are due to the complications arising from metastasis. According to cancer statistics, approximately 60% of colorectal cancer patients are expected to develop metastasis. Although several approachs for treatment have been developed recently for colorectal cancer, patients with advanced or metastatic colorectal cancer still have poor prognosis. In this study, we showed that Cyclin B1 suppressed colorectal cancer invasion and metastasis through regulation of E-cadherin expression.

The regulation of orderly cell cycle progression is essential for cells to maintain genomic integrity which is vital for cell survival and controlled proliferation. Cyclins are a family of proteins that vary in their expression levels during the cell cycle to activate their specific cyclin-dependent kinases required for progression through the cell cycle. Among them, Cyclin B1 is of paramount interest because it was originally believed that Cyclin B1 controlled G2-M phase checkpoint and regulated the correct onset of mitosis, which are both essential for DNA synthesis and cell proliferation. Accumulated evidences have showed that Cyclin B1 were overexpressed in breast cancer, esophageal squamous cell carcinoma, lung cancer, gastric cancer, hepatocellular cancer, and other cancer cells [31, 32, 41–43]. Statistical evidence suggested that Cyclin B1 was related to tumor malignance. In addition, the overexpression of Cyclin B1 has been identified as a promising poor prognostic marker in patients with hepatocellular cancer, non-small cell lung cancer, and head-neck squamous cell carcinoma [32, 34, 43]. However, Chae et al. [44] reported that Cyclin B1 expression had no influence on the survival of patients with breast cancer. Bjorck et al. [38] reported that patients with follicular lymphoma expressing higher level of Cyclin B1 showed better outcome after chemotherapy, compared to patients expressing lower level of Cyclin B1. These discordant findings may be explained by the dissimilar expression pattern of Cyclin B1 in different tumor types. We previously found that Cyclin B1 was overexpressed and important for cell proliferation and tumor growth via promoting cell cycle progression and/or reducing apoptosis in colorectal cancer cells [37]. However, the role and mechanism of Cyclin B1 in colorectal cancer metastasis has not been well studied.

In this study, we found Cyclin B1 expression level in colorectal cancer tissues was also higher than that in nomal tissues in a large panel of 150 patients, consistent with our previous data. However, Cyclin B1 overexpression was negatively correlated with aggressive clinicopathological features, such as lymph node metastasis, distant metastasis, and TNM stage. Patients who had low expression of Cyclin B1 were remarkably poor overall survival compared with patients who had high expression of Cyclin B1. Univariate analysis demonstrated that Cyclin B1 was a prognostic indicator for overall survival in patients with colorectal cancer, though multivariate analysis showed Cylcin B1 was not an independent prognostic factor. Taken together, these results clearly demonstrated that low Cyclin B1 level is associated with poor prognosis and unfavorable clinical outcome of colorectal cancer. However, our results are not in agreement with other two studies [35, 45]. Heike et al. showed Cyclin B1 overexpression was neither associated with histopathologic tumor features nor poor prognosis in colorectal cancer, whereas Jia-Qing et al. revealed Cyclin B1 overexpression intensified during carcinogenesis, but abated during invasion, and then increased again during metastasis in colorectal cancer.

To further support our notion, the role and mechanism of Cyclin B1 in metastasis were detected in different colorectal cancer cell models, p53^+/+^ HCT116, p53^-/-^ HCT116, and SW480. We provided evidence that knockdown of Cyclin B1 expression induced more cells to infiltrate through the matrigel and transwell membrance in all three cancer cell lines, demonstrating that Cyclin B1 suppresses colorectal cancer metastasis by negatively regulating cell migration and invasion. EMT had been implicated as the key step in the progression of tumors toward invasion and metastasis [9]. E-cadherin was a well-known cell-cell adhesion molecule, which formed epithelial adherent junctions and sequestrated CTNNB1. In the EMT changes, suppression of E-cadherin is a crucial step during the progression of many cancers including colorectal cancer [15, 46]. It will be of particular importance to explore the underlying mechanism that result in E-cadherin suppression. Despite many molecules have been identified to modulate E-cadherin expression [17, 47, 48], more studies are still needed to elucidate the important signaling molecules for regulating E-cadherin. Here, we demonstrated that Cyclin B1 participated in the regulation of E-cadherin expression. Inhibition of Cyclin B1 downregulated E-cadherin protein in p53^+/+^ HCT116, p53^-/-^ HCT116, and SW480 cells. In addition, in vivo xenograft assays also showed E-cadherin level was significantly decreased in Cyclin B1-suppressing p53^+/+^ HCT116 and SW480 tumors, compared to respective control tumors. Thus, we speculate that Cyclin B1 may suppress colorectal cancer invasion and metastasis by inducing E-cadherin expression. However, further experiments still need to be done to clarify the mechanic link between Cyclin B1 and E-cadherin.

On the basis of our previous published data and our current observations, we suggest that Cyclin B1 is overexpressed and promotes cell proliferation during the early stage of colorectal cancer development. When tumor cells invade into surrounding tissues and further metastasize into distant tissues, the expression of Cyclin B1 is decreased and negatively correlated with poor survival. Further function study showed suppression of Cyclin B1 could promote tumor cell migration and invasion and reduce E-cadherin expression. Moreover, we also confirmed the effect of Cyclin B1 on E-cadherin in vivo mice tumor model. Thus, similar as other papers [49, 50], our results suggest Cyclin B1 favors tumor growth but inhibits metastasis in colorectal cancer.

In summary, this study provides new insight into the role of the Cyclin B1 in colorectal cancer metastasis. Our finding that the low level of Cyclin B1 expression promoted colorectal cancer metastasis by regulating E-cadherin highlights the potential of Cyclin B1 as a novel agent against colorectal cancer metastasis.

## Supporting Information

S1 Fig Ethics statements of human tissue samples. The study of human colorectal tissues was approved by the Sir Run Run Shaw Hospital Research Ethical Committee for clinical research.
(TIF)

S1 Table Clinical information of 150 patients with colorectal cancers. (XLS)
