# Supplementary figures and images for: Cyclin B1 Suppresses Colorectal Cancer Invasion and Metastasis by Regulating E-Cadherin
Source: PLoS One. 2015 May 11;10(5):e0126875. doi: 10.1371/journal.pone.0126875 (PMC4427130; doi:10.1371/journal.pone.0126875)

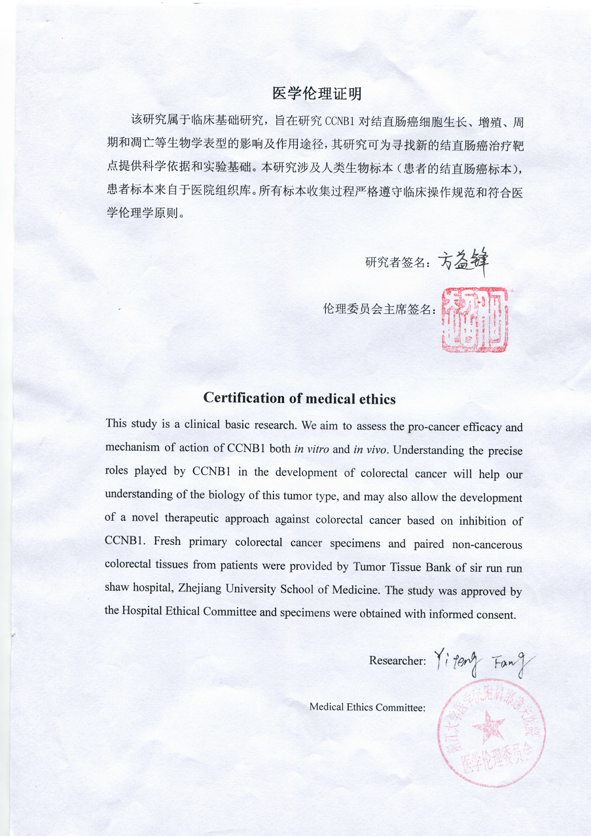

Supplement: S1 Fig — The study of human colorectal tissues was approved by the Sir Run Run Shaw Hospital Research Ethical Committee for clinical research. (TIF) [file pone.0126875.s001.tif]
